# Fire and Snow: Effects of Snowpack Variation and Wildfire on Small Mammal Dynamics in Sub‐Alpine Habitats

**DOI:** 10.1002/ece3.73525

**Published:** 2026-04-20

**Authors:** Ken P. Green, David C. D. Happold, Glenn M. Sanecki, Christopher R. Dickman

**Affiliations:** ^1^ College of Asia and the Pacific The Australian National University Canberra Australian Capital Territory Australia; ^2^ Research School of Biology The Australian National University Canberra Australian Capital Territory Australia; ^3^ NPWS Jindabyne New South Wales Australia; ^4^ School of Life and Environmental Sciences The University of Sydney Sydney New South Wales Australia

**Keywords:** predation, small mammals, snowpack, sub‐alpine, wildfire

## Abstract

Montane ecosystems in many parts of the world are threatened by climate change. In Australia, alpine and sub‐alpine habitats face warming temperatures, increased risk of wildfire and incursions by invasive species. For small mammals that are active over winter in these habitats, a further risk may arise if the winter snowpack—and the thermally buffered subnivean space that it creates—is impacted. We examine long‐term (19–43‐year) datasets on three species of small mammal in sub‐alpine habitats in Australia to test whether populations decline in years when there is a shallow or transient snowpack, and after wildfire. We anticipated that species would show differential susceptibility to snowpack integrity, with species such as the broad‐toothed rat ( *Mastacomys fuscus* ), which nests on the soil surface within the subnivean space, likely to be at most risk in years of shallow or transient snow cover. We found that populations of *M. fuscus* often declined following winters with shallow snow cover, and that the end of snow cover influenced numbers of *M. fuscus* and a second species, the mainland dusky antechinus (*Antechinus mimetes*) in the following summer or autumn. Populations of a third study species, the bush rat ( *Rattus fuscipes* ), were less affected by snowpack variables. All species declined sharply post‐fire; depressions in numbers were most prolonged, and recoveries slowest, for *A. mimetes* and particularly *M. fuscus* . Food availability, drought, vegetation cover and differential susceptibility of the study species to predation by the European red fox ( *Vulpes vulpes* ) probably contributed to the observed dynamics. We suggest that interactions between snowpack reduction, wildfire and fox predation pose increasing threats to small mammals in Australia's sub‐alpine habitats, and call for the development of local management plans for taxa at most risk of collapse.

## Graphical abstract

Global warming is increasing the risk of fire and decreasing winter snow cover in montane environments in many parts of the world. Using a long term trapping study in sub‐alpine habitats in the Australian Alps, we show that changes in the fire regime and snow conditions interact with other factors (availability of food and predation by introduced carnivores) to threaten populations of three species of endemic small mammals.

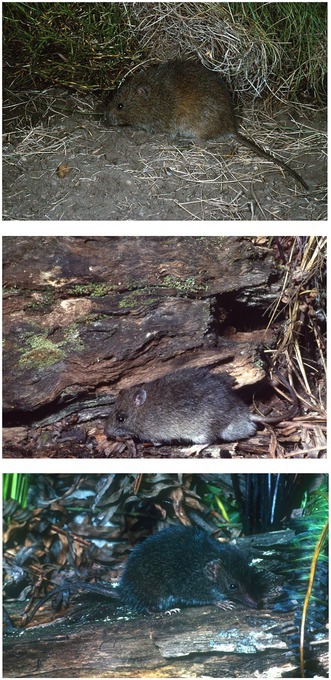

## 1 Introduction

Disturbances of anthropogenic origin are affecting biota in many parts of the world. Some species are adapting to these disturbances and the new habitats they create (Otto 2018), but many are unable to cope and face local or regional extinction (Kelly et al. 2020; Ceballos and Ehrlich 2023). Over‐harvesting species from the wild, land clearing and invasive species are the most prevalent disturbances for currently threatened or near‐threatened species (Maxwell et al. 2016); these and other threats often interact to deplete populations at different spatial scales (Doherty et al. 2015; Greenville et al. 2021). Climate change was considered by Maxwell et al. (2016) to threaten relatively few species, but recent studies report extinctions that are due directly to this threat (Waller et al. 2017; Urban 2024); more extinctions are likely as climate impacts intensify (Olivares‐Rojas et al. 2024; Ripple et al. 2024).

Among the environments likely to be most affected by climate change are alpine and sub‐alpine ecosystems (Urban 2024). Here, warming temperatures and changes in rainfall will push many species and ecological communities beyond their bioclimatic limits, while also increasing risks of wildfire and ingress of invasive species and pathogens (Grabherr et al. 2010; McDougall et al. 2018). Increased disturbance from activities such as high‐altitude agriculture, tourism, development and roading will likely exacerbate these effects, leading to fragmentation of the ranges of some species and to local extinctions of others (Williams et al. 2014; Tolvanen and Kangas 2016). Many species on mountain tops have narrow ranges and will be especially difficult to conserve elsewhere (Noroozi et al. 2018; Vintsek et al. 2022).

Small mammals are of particular conservation concern in many alpine regions as they cannot move to other environments and are susceptible to genetic erosion as their numbers decline (Martin 2001; Rubidge et al. 2012). Populations of small mammals in some alpine regions, such as the European Alps, are also poorly understood (Allainé and Yoccoz 2003), in part because they are hard to access under snow and where there is risk of avalanches. However, as alpine areas can provide relatively predictable climates for small mammals (Yoccoz and Ims 1999), some species show regular population fluctuations or even stability (Merritt 2010; Krebs 2013). For example, survival rates of voles ( *Myodes glareolus* ) in the French Alps vary little between years despite annual differences in depth and duration of snowcover (Yoccoz and Mesnager 1998). In the Australian Alps, Happold (2015) reported moderate inter‐annual fluctuations in population size of small mammals, as did Rudá et al. (2010) for a 12‐year study of the Tatra vole ( *Microtus tatricus* ) in the Western Tatra Mountains in Slovakia.

In Australia, alpine and sub‐alpine areas occur in the Australian Alps bioregion in the south‐east of the continent. This bioregion covers 12,330 km^2^ with 1877 km^2^ defined as alpine and subalpine (Green and Stein 2015), and contains many threatened plants, vegetation communities and threatened vertebrates (Green and Pickering 2002; Green and Osborne 2012). Although the highest peak, Mt. Kosciuszko, is only 2228 m, the Australian Alps maintain a ‘high mountain’ environment (*sensu* Körner and Spehn 2002). The period of snow cover ranged historically from 30 days at the winter snowline (Costin 1954) to ~120 days above the treeline (Slatyer et al. 1984), but snow accumulation, maximum snow depth and persistence have declined over the last 50 years due to accelerated warming, with snow cover now at its lowest for at least 2000 years (McGowan et al. 2018; Bureau of Meteorology and CSIRO 2024). Climate modelling predicts that these trends will continue; by 2050, the area supporting snow in the Australian Alps for more than 60 days a year may be reduced by up to 96% (Hennessy et al. 2003). These changes, and increased incidence of wildfires, are likely to profoundly affect vegetation (Hoffmann et al. 2019; Morgan and Walker 2023) and have negative effects on small mammals that depend on intact vegetation and a deep and persistent snowpack for over‐winter persistence (Happold 1989, 1998; Sanecki et al. 2006).

Five species of native small mammal occur above the winter snowline in Australia. The mountain pygmy‐possum ( *Burramys parvus* ), a Critically Endangered species restricted to alpine and sub‐alpine habitats (IUCN 2023), hibernates over winter and depends on an intact snowpack to maintain stable conditions (Broome et al. 2012). Two species of dasyurid marsupial also occur. The mainland dusky antechinus (*Antechinus mimetes*), referred to as *A. swainsonii* in earlier publications (e.g., Dickman 1982; Green 1998; Happold 1989, 2015), is common above the treeline; it uses the subnivean space during winter to hunt invertebrates, and shelters in shallow burrows (Sanecki et al. 2006). The agile antechinus ( *A. agilis* ) occurs in the low sub‐alpine where it nests in trees (Green 1998), but is rare at the highest treeline (Green and Osborne 2012). The bush rat ( *Rattus fuscipes* ) and broad‐toothed rat ( *Mastacomys fuscus* ) are active year‐round in the sub‐alpine and alpine zones; *R. fuscipes* has a plant and fungus‐based diet and digs deep but twisting burrows in soil to depths of > 78 cm to escape winter conditions (Warneke 1971; Stewart 1979), whereas *M. fuscus* is herbivorous and in winter constructs large, well‐insulated grass nests amidst vegetation on the soil surface (Green et al. 2014). *Rattus fuscipes* and the *Antechinus* species exploit varied heathland and forest habitats in eastern Australia, but *M. fuscus* in New South Wales occurs at altitudes mostly > 1000 m (Green and Osborne 2003; Belcher and Leslie 2011). It is near threatened (IUCN 2023) and is projected to decline further due to climate change (Green et al. 2008).

In view of the stability that usually characterises the dynamics of alpine small mammals, the rapid decline of species like *M. fuscus* since European settlement in Australia is of concern (McDowell et al. 2023). Grazing, particularly by cattle, rabbits ( *Oryctolagus cuniculus* ) and feral horses ( *Equus caballus* ) degrades habitat and has probably led to losses of *M. fuscus* at altitudes up to the sub‐alpine zone (Belcher and Leslie 2011; Schulz et al. 2019). Increased incidence of wildfire below the treeline is likely also to reduce habitat suitability for small mammals (Driscoll et al. 2025), although impacts on some species—including *M. fuscus* —can be short‐term (Dickman and Happold 2022; Schulz et al. 2024). Predation by European red foxes ( *Vulpes vulpes* ) is a threat at all altitudes, with foxes preying more heavily on *M. fuscus* than on other sympatric mammals (Green and Osborne 1981; Green 2002) and preventing successful dispersal of *M. fuscus* between habitat patches (O'Brien et al. 2008).

**FIGURE 1:**
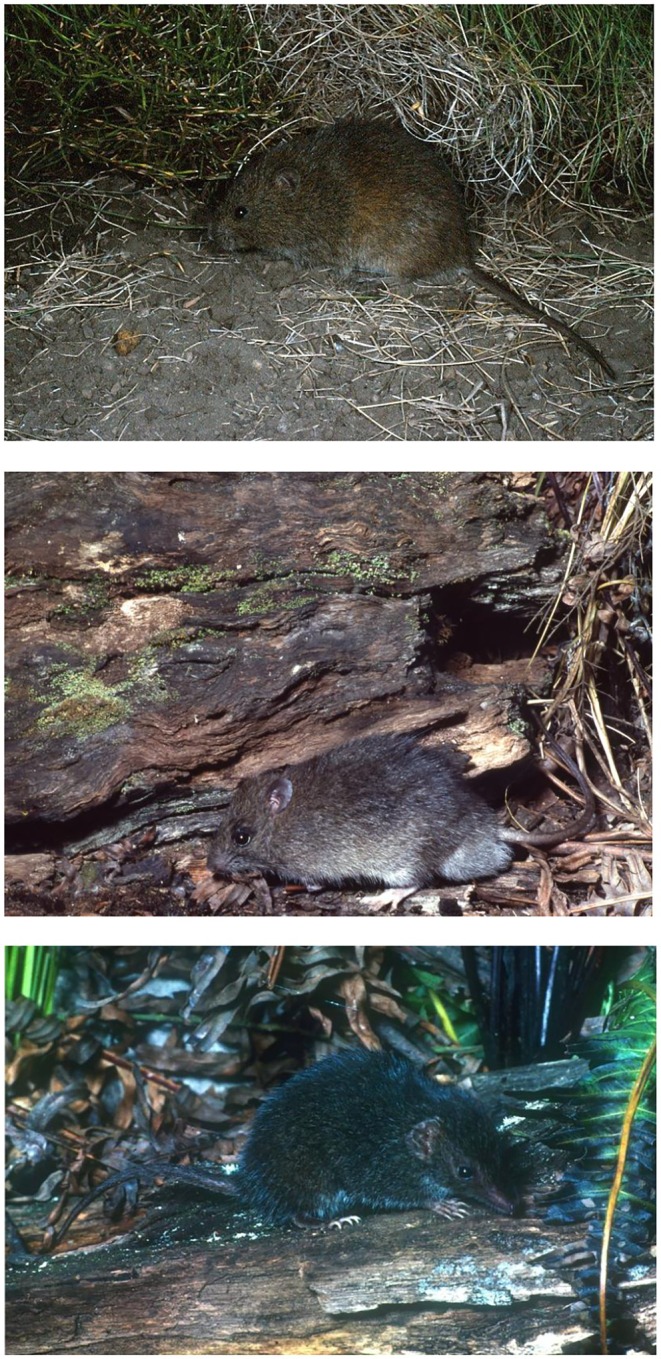
The three study species. Top, the broad‐toothed rat ( *Mastacomys fuscus* ); middle, bush rat ( *Rattus fuscipes* ); bottom, mainland dusky antechinus (*Antechinus mimetes*). Photos: *M. fuscus* —David Happold; *R. fuscipes* and *A. mimetes* —Christopher Dickman.

In this study, we examine a 19–43‐year dataset on *M. fuscus* , *R. fuscipes* and *A. mimetes* (Figure 1) at sub‐alpine sites in the Australian Alps and investigate factors that may drive population declines. Based on the above considerations, we propose three hypotheses:

1. Populations will decline in years when there is a shallow or transient snowpack,
2. Declines will be most severe for *M. fuscus* and less severe for *R. fuscipes* and *A. mimetes* owing to the greater exposure of the nests of *M. fuscus* to extreme climatic conditions and potential fox predation when the snowpack is shallow or transient, and
3. Populations of all species will decline sharply after wildfire.

Other factors that cause declines of these species elsewhere, including trampling by feral horses and potential competitors such as the swamp rat (*Rattus lutreola*) (Green et al. 2008; Schulz et al. 2019) are not considered further as they were not present, or were very scarce, in our study area. Introduced fallow deer ( *Dama dama* ) and sambar deer ( *Cervus unicolor* ) were not present for most of the study period, but arrived and established populations from about 2015 (K. Green unpub.), and are now present in many areas (McCarthy et al. 2025).

## 2 Materials and Methods

### 2.1 Study Area

**FIGURE 2:**
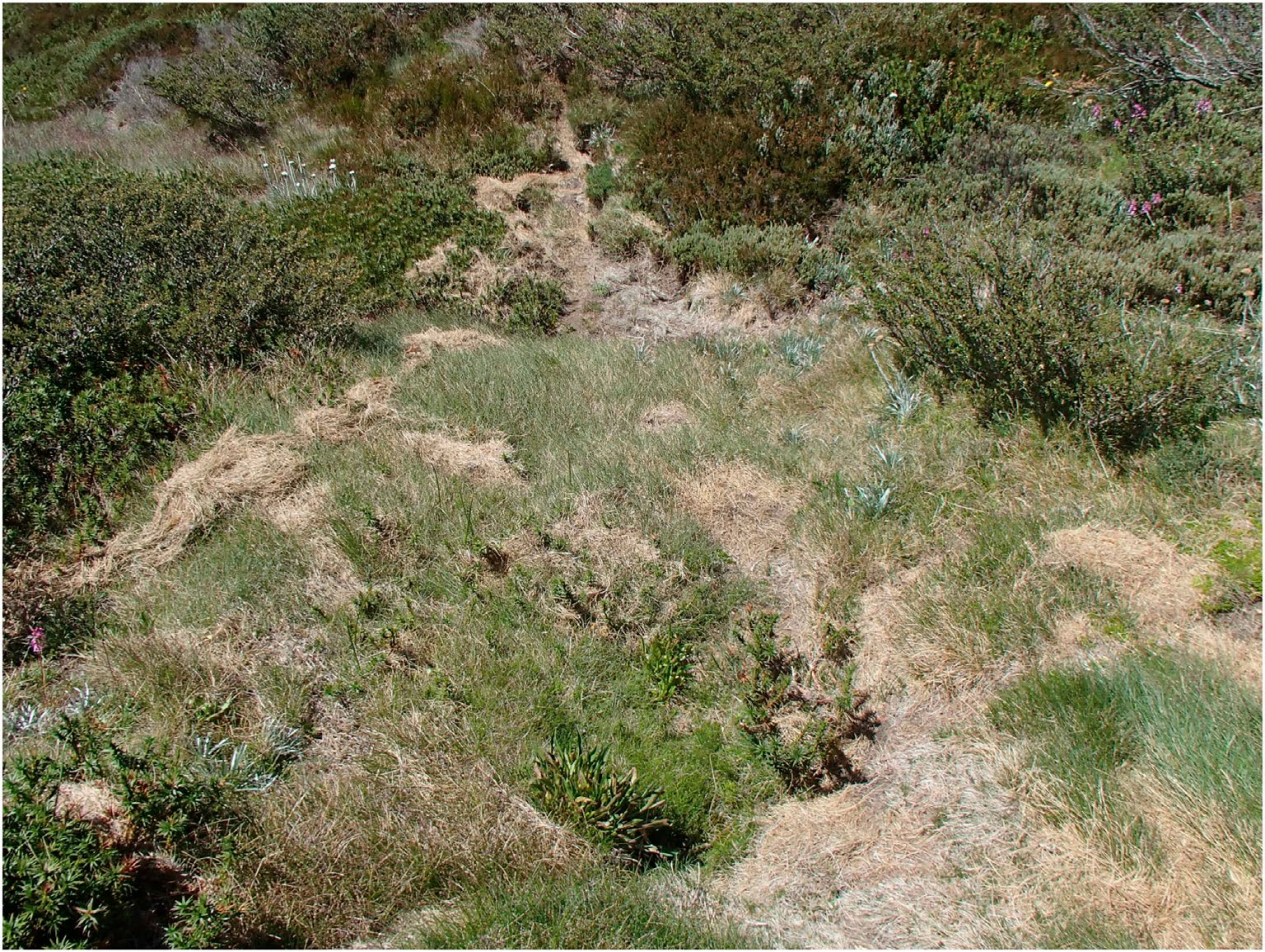
Small mammals that are active in the subnivean space over winter can leave signs of their activity following snowmelt in spring. Here, broad‐toothed rats ( *Mastacomys fuscus* ) have created runways and winter nests, cutting and collecting *Poa* grass which is stored for food beneath the snowpack. Photo: Ken Green.

Our main study site, ~1.7 ha, was located in a valley near Smiggin Holes in Kosciuszko National Park (36°24′ S, 148°26′ E) at an altitude of ~1680 m. The site extends along a tributary of Pipers Creek (Happold 2015) and is dominated by wet heathland comprising *Bossiaea foliosa* and species of *Hovea*, *Kunzea*, *Callistemon*, *Prostanthera*, *Nematolepis* and *Leptospermum*, with an understorey of *Poa* grass (Happold 2015). Patches of sphagnum bog and snowgum *Eucalyptus niphophila* woodland also occur. Mean monthly temperatures at a nearby weather station (Perisher Valley, 1735 m) peak in January (12°C) and are lowest in July (−1.4°C); mean annual rainfall is 1950 mm, with ~50% of this falling as snow in winter (Happold 1998). The annual snowpack lasts for 17–25 weeks and ranges in depth from 90–350 cm; importantly, it insulates the soil and subnivean space so that small mammal runways and burrows remain at −1°C to 0°C (Happold 1998, 2015; Figure 2). A wildfire burnt this site in the summer of 2002–2003. Annual rainfall also was reduced during the Millenium Drought between 2001 and 2009 (1337.5 mm/year compared with the annual average rainfall of 1912.8 mm/year between 1977 and 2000) (Green and Sanecki 2006; van Dijk et al. 2013).

Several secondary study sites were established in 2002 to assess whether the small mammal population dynamics at our main site occurred generally across the sub‐alpine environment. These sites, all in floristically and topographically similar locations to the main site, were at Horse Camp (36°20′ S, 148°24′ E; 1540 m), Perisher Creek (36°23′ S, 148°24′ E; 1620 m), Rainbow Lake (36°22′ S, 148°29′ E; 1600 m), Whites River (36°18′ S, 148°23′ E; 1620 m) and Kerries Ridge (36°15′ S, 148°25′ E; 1780 m). Each site covered ~1 ha and was located 2.1–16.7 km (mean = 8.42 ± 5.82 km SD) from the main site. Some sites had been used earlier by Calaby and Wimbush (1964), Carron (1985), Belcher (1988) and Hill (1996). Except for Horse Camp and Perisher Creek, all sites experienced wildfire in early 2003.

### 2.2 Field Sampling

Small mammals were censused at all sites using grids of Elliott box traps (30 × 10 × 9 cm). At the main site 12–15 traps were set along lines 15 m apart either side of the creek, with traps spaced 15 m apart on the lines. One line had nine additional traps spaced 5 m apart that ran along a small water course (Bubela et al. 1991). The position of the grid was adjusted slightly in 2018 to reduce nearby road disturbance. Traps were placed in plastic bags (20 × 30 cm) to keep them dry, provided with a bait of peanut butter, rolled oats and honey, bedding of non‐absorbent cotton wool or coconut fibre, and set for three consecutive nights 1–3 times a year in spring (early December), summer (February) and autumn (April). Traps were checked after dawn and in the afternoon each day and captured animals were identified, weighed, checked for sex, age (young of the year or adult) and reproductive condition, then released (Happold 2015). Animals were marked individually; this initially entailed removal of a terminal toe phalange, but later for *R. fuscipes* and *A. mimetes* we used ear tags (Hauptner Australia, cat. no. 73850) or ear punches. From 1997 onwards, *M. fuscus* was marked by microchipping (Trovan, ID100VB, Keysborough, Victoria). Traps were cleaned and reset each trapping session and removed on the final day. Trapping at the secondary sites followed the same protocols as at Smiggin Holes but was carried out only during February or April.

Trapping at the main site was carried out in most years from summer 1978–1979 until 2020. No data were recorded for *A. mimetes* or *R. fuscipes* in 1978, and no trapping occurred in 1981, 1992–1995 and 1997. Trapping in spring, summer and autumn continued until 2008. Spring trapping ceased after 2008, but trapping continued in February or April for the remaining years of the study. Trapping at all the secondary sites continued until 2020.

To gauge red fox activity, we used two methods. First, we counted fox tracks after fresh snow falls in winter from 1996 to 2005 along a ski transect near Perisher Valley (1.7 km). All fox tracks that crossed the transect were counted, but if a track criss‐crossed the transect it was counted only once. Second, each month from 1996 to 1998, and then opportunistically until 2004, we counted fox scats while walking along a four‐wheel drive track, clearing the track on each occasion. The track was 2–3 m wide and ran for ~7 km at altitudes of 1500–1600 m (Green 2003). To account for differences in effort expended on the two methods, we standardised the results as average numbers of tracks or scats recorded per month.

Following the wildfire in early 2003, the cover of shrubby vegetation was severely reduced and unlikely to support a snowpack or subnivean space. To quantify vegetation recovery, we used point intercepts to record vegetation coverage at five points around each grid; one point was located centrally in the grid and four points were located within 3 m of the grid edges. At each point we used a pole and scored the presence (1) or absence (0) of vegetation at 20 cm and in increments of 20 cm up to 120 cm, with the final record being of any vegetation > 120 cm high. Vegetation surveys began in late 2003, 9 months post‐fire, and were repeated in 2005, 2007 and then yearly until 2016. Scores were averaged for each grid, and across grids, each year, and expressed as the proportion of vegetation‐presences at each height class.

### 2.3 Environmental Variables

Snowpack duration and depth data were obtained from the Snowy Hydro snow course at Deep Creek at an altitude of 1620 m (https://www.snowyhydro.com.au/generation/live‐data/snow‐depths/, accessed 3 February 2024). Snow data have been recorded here weekly throughout winter since 1957. The altitude of Deep Creek is similar to our study sites, and our on‐site observations of the start and end of snow cover at our sites accorded closely with the respective dates recorded at Deep Creek. The beginning of the snow season was taken as the date midway between the last zero reading at Deep Creek and the first reading of snow depth leading to the establishment of a continuous winter snow pack. The end of the snow season was taken as the date when the snow course at Deep Creek recorded no snow.

### 2.4 Data Analysis

Estimates of population size used trapping results from February or April each year at all sites and were expressed as ‘minimum numbers of animals known to be alive’ (MNA, Krebs 1966). We focussed on February–April because young of the year—especially *A. mimetes* —were trappable then and this period provided the longest time series of data at the main site. We also calculated the rate of increase (Caughley and Sinclair 1994; Dickman et al. 2010) for each species as the difference in MNA from 1 year to the next, and expressed this as the rolling average for adjacent 3‐year periods to visualise periods of major population change. We checked for temporal autocorrelation in MNA at the main site from 1998 to 2020 and at the secondary sites from 2002 to 2020 as these were the longest continuous runs of annual data (i.e., no consecutive years were missing), and also exceeded the minimum 15‐year period defined by Swanston (1998) to reliably detect autocorrelation if it is present. The results revealed no evidence of significant autocorrelation: for lags of 1–8 years at the main site, autocorrelation averaged −0.02 for *M. fuscus* , −0.15 for *R. fuscipes* and −0.12 for *A. mimetes* . At the unburnt secondary sites autocorrelation averaged −0.06 for *M. fuscus* , −0.05 for *R. fuscipes* and −0.05 for *A. mimetes* ; at the burnt sites respective values for these species were −0.1, 0.13 and −0.17.

To explore snowpack effects on small mammals, we derived a series of variables describing annual snow depth and duration. Using the Deep Creek records, these variables were: max. snow depth (cm), mean snow depth (cm, based on week by week depth measurements during the period of snow cover), the coefficient of variation of mean snow depth, days in the calendar year when the snow season began and ended (i.e., Julian days) and the seasonal duration of snow cover (excluding any periods during the season when snow cover was zero) (Table S1). A correlation matrix, based on Pearson's *r*, showed that some of these variables were correlated with each other (*r* > 0.7, *p* < 0.001). To reduce autocorrelation, we selected three variables: the start of the snow season (calendar days), the end of the snow season (calendar days) and the product of mean snow depth and seasonal duration (cm days). Correlations between these variables ranged from 0.03 to 0.66. At the main study site, annual trapping results indicated that populations of all species declined to zero for one or more years following the wildfire in early 2003. We depict results for these years (2003–2006), but exclude them from analyses of the effect of snowpack on small mammals. Snowpack data were also excluded from analyses for years when no trapping was undertaken (1981, 1992–1995 and 1997). At the secondary sites we averaged the numbers of animals captured each year in the two unburnt sites (Horse Camp and Perisher Creek) separately from those captured in the three burnt sites (Rainbow Lake, Whites River and Kerries Ridge).

Any effects of the winter snowpack on small mammals were expected to occur during or after the period of snow coverage; hence, we assumed that the strongest signal of any effect would be detectable in our trapping results in the following summer. To account for this, and test our first hypothesis, we compared our estimates of animal numbers each February (Table S2) with snowpack variables from the previous winter. We used lagged MNA for each species as the dependent variable in multiple regression analyses, with start and end of the snow season and the product of mean snow depth and seasonal duration as independent variables. Rates of population increase were used as the dependent variable in further regressions, but were not lagged as population changes occurred between trapping sessions, coincident with the measured snow variables. To test our second hypothesis, we computed analyses of covariance to compare regression slopes for MNA and rate of increase for the three species against each separate independent variable at the main study site (Quinn and Keough 2002). To test our third hypothesis, animal numbers in the burnt and unburnt sites were compared using repeated measures analyses of variance. Prior to analyses, we checked variables for normality and equality of variances using Shapiro‐Wilks and Levene's tests. The raw product of mean snow depth and seasonal duration was skewed (*W* = 0.91, *p* = 0.002) but was normalised by log_10_ transformation (*W* = 0.97, *p* = 0.259), so we used cm days' in analyses. Excel was used for univariate analyses and Genstat (version 14.2.0.6297) for all others. Statistical significance is accepted at *α* ≤ 0.05 and is denoted by * unless exact *p*‐values are shown.

## 3 Results

**FIGURE 3:**
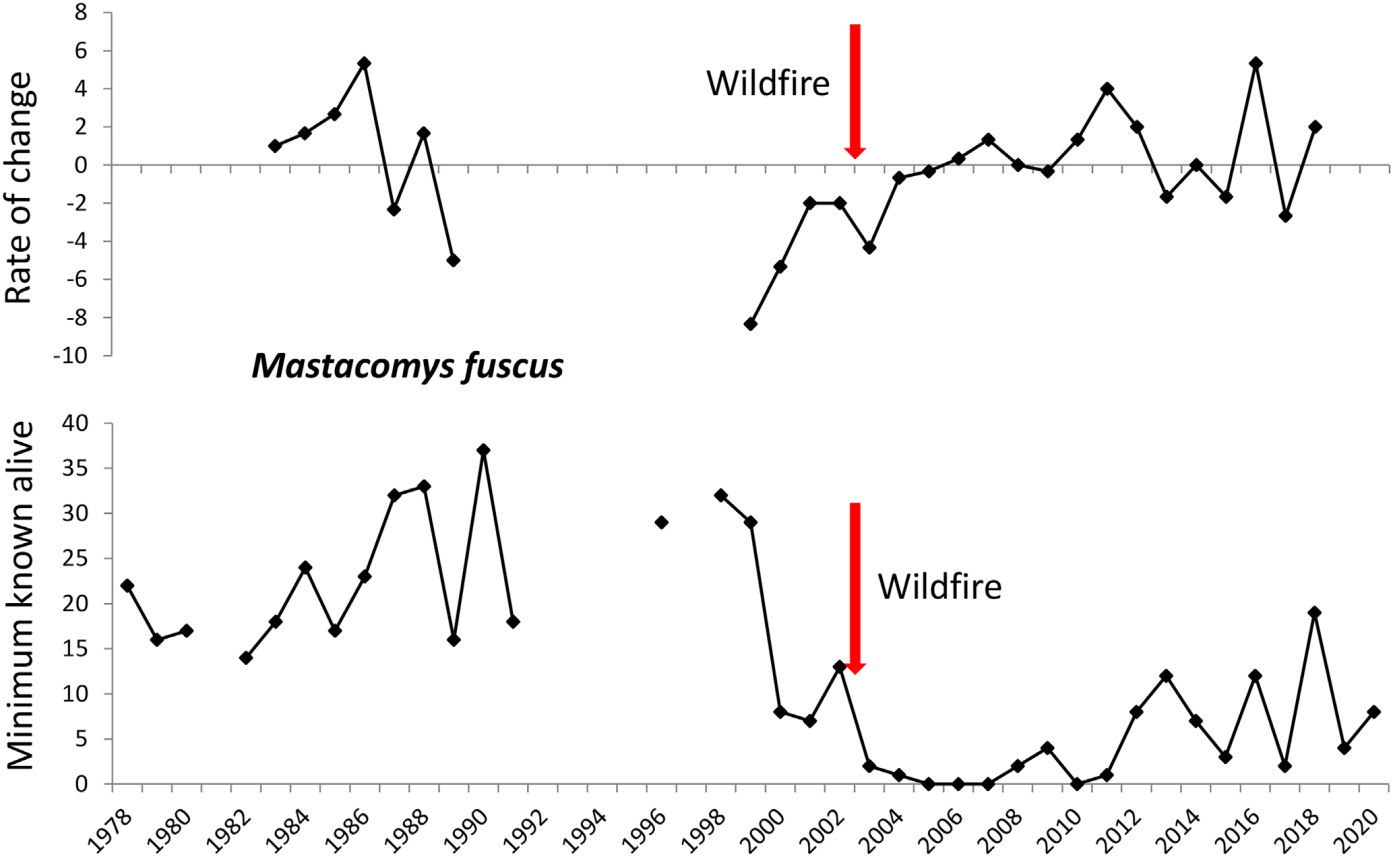
Demographic changes of the broad‐toothed rat *Mastacomys fuscus* between 1978 and 2020 at the main study site at Smiggin Holes, Kosciuszko National Park. Rate of change in numbers is shown in the top panel, expressed as the rolling average for adjacent 3‐year periods, and minimum number of animals known alive is shown in the bottom panel. A wildfire burnt most of the site in early 2003, denoted by the red arrow.

**FIGURE 4:**
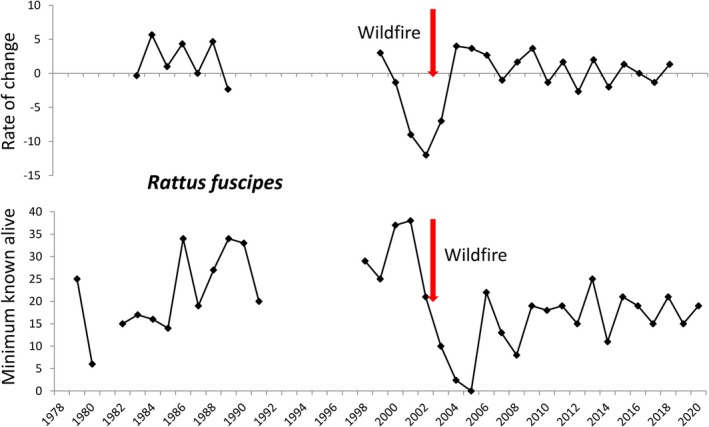
Demographic changes of the bush rat *Rattus fuscipes* between 1979 and 2020 at the main study site at Smiggin Holes, Kosciuszko National Park. Rate of change in numbers is shown in the top panel, expressed as the rolling average for adjacent 3‐year periods, and minimum number of animals known alive is shown in the bottom panel. A wildfire burnt most of the site in early 2003, denoted by the red arrow.

**FIGURE 5:**
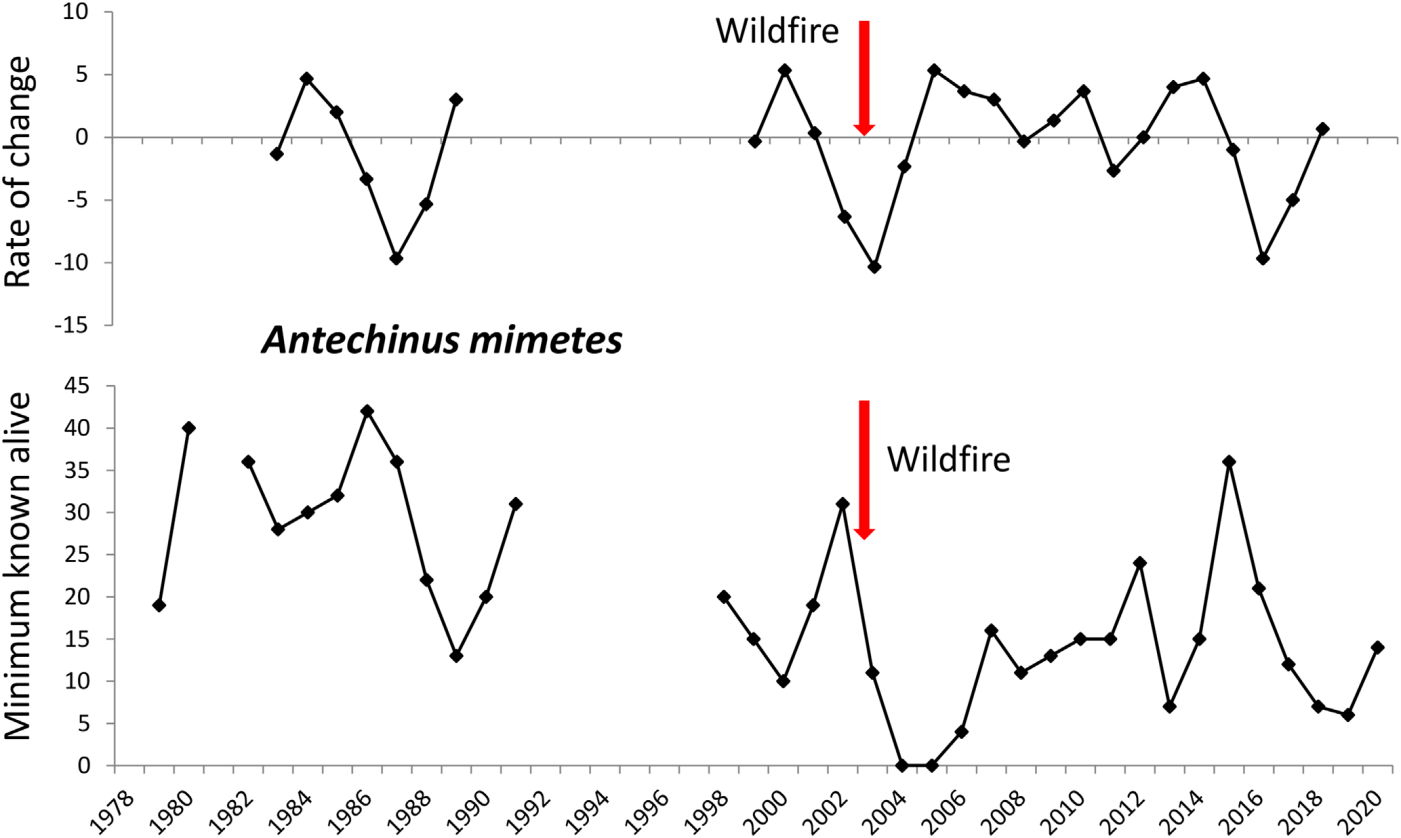
Demographic changes of the mainland dusky antechinus *Antechinus mimetes* between 1979 and 2020 at the main study site at Smiggin Holes, Kosciuszko National Park. Rate of change in numbers is shown in the top panel, expressed as the rolling average for adjacent 3‐year periods, and minimum number of animals known alive is shown in the bottom panel. A wildfire burnt most of the site in early 2003, denoted by the red arrow.

**FIGURE 6:**
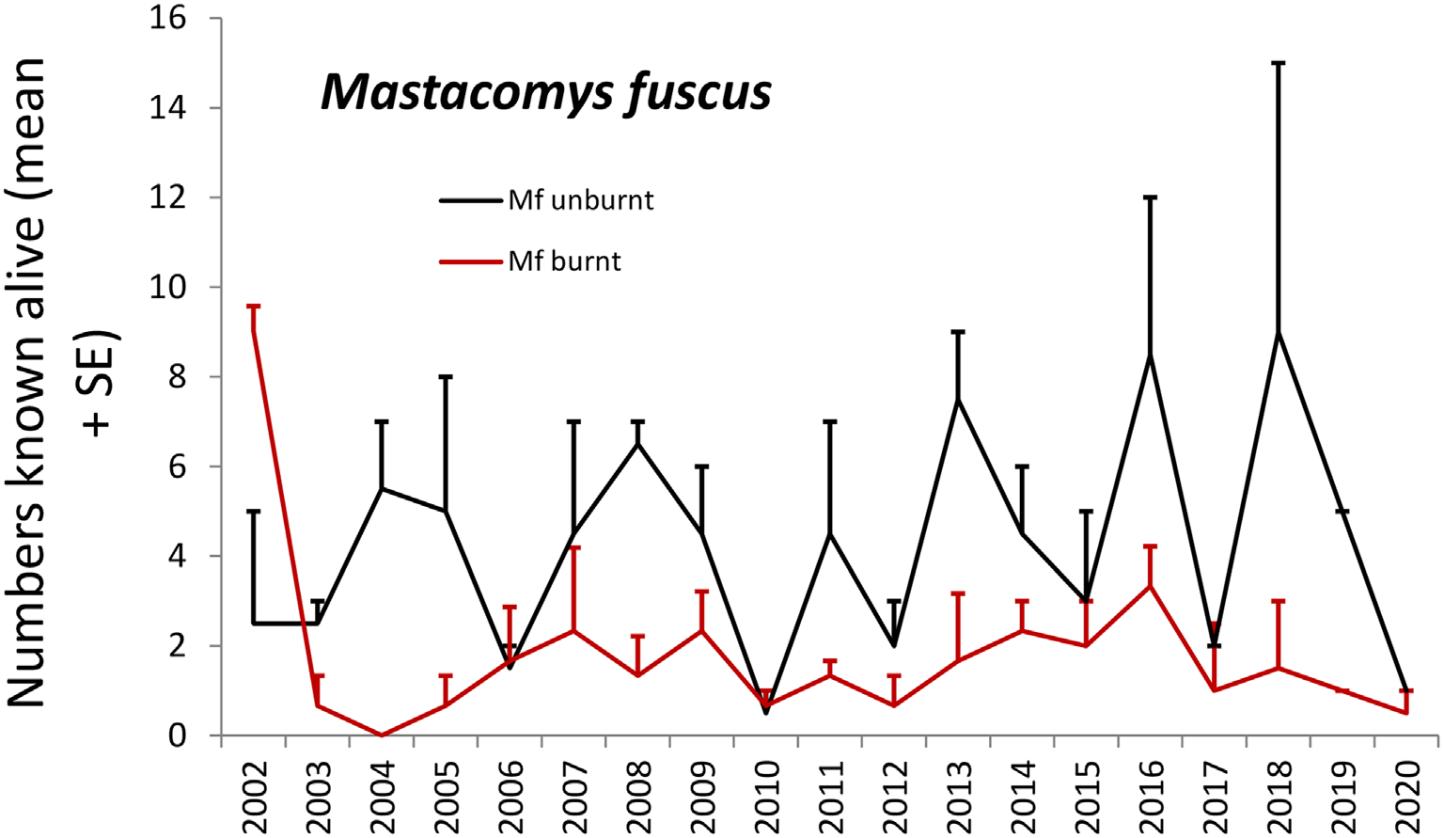
Numbers of broad‐toothed rats *Mastacomys fuscus* known alive between 2002 and 2020 at secondary study sites in Kosciuszko National Park, expressed as means + SE for two unburnt sites (Horse Camp and Perisher Creek, black line) and three sites (Rainbow Lake, Whites River and Kerries Ridge, red line) that burnt in early 2003.

**FIGURE 7:**
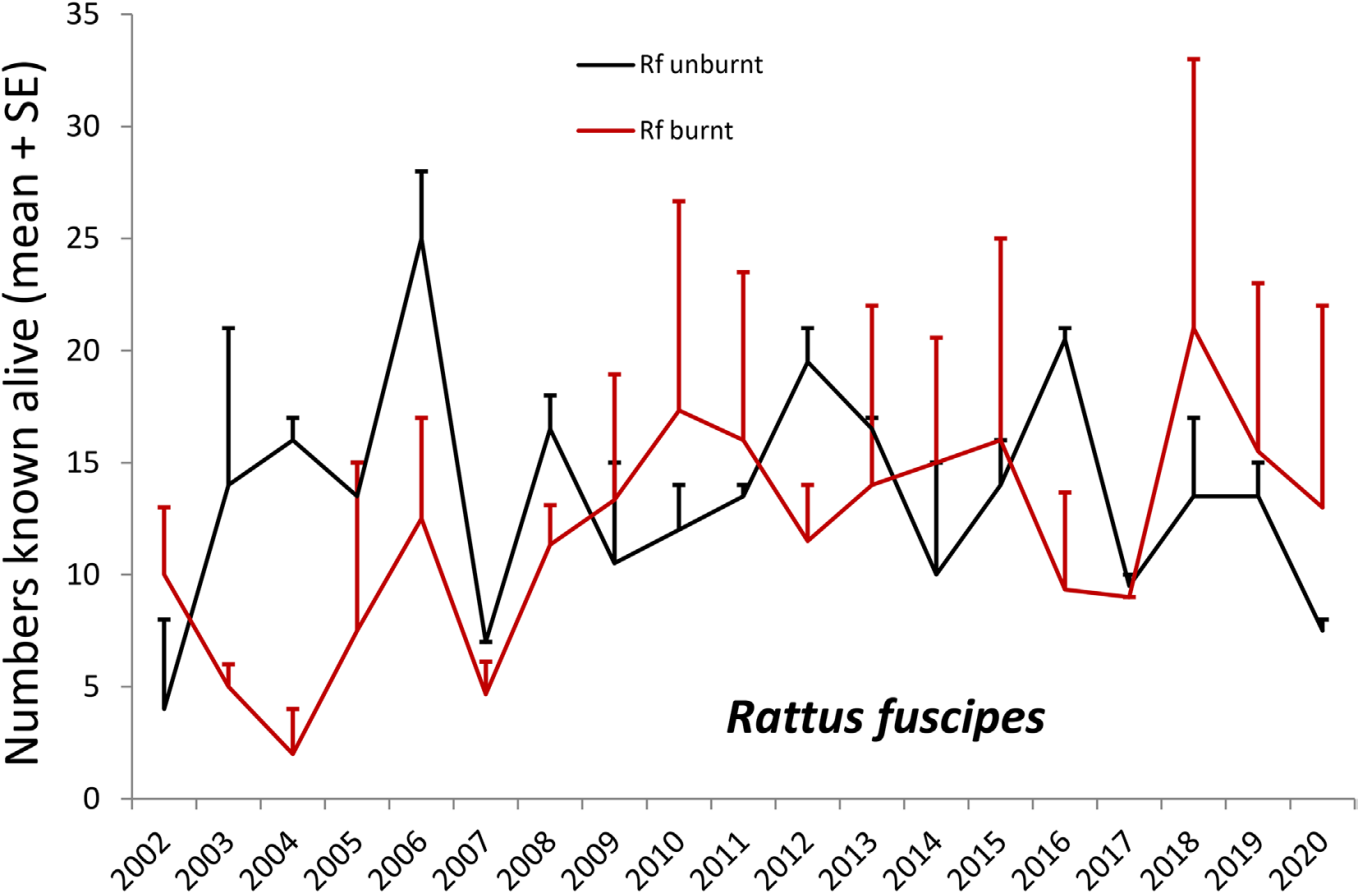
Numbers of bush rats *Rattus fuscipes* known alive between 2002 and 2020 at secondary study sites in Kosciuszko National Park, expressed as means + SE for two unburnt sites (Horse Camp and Perisher Creek, black line) and three sites (Rainbow Lake, Whites River and Kerries Ridge, red line) that burnt in early 2003.

**FIGURE 8:**
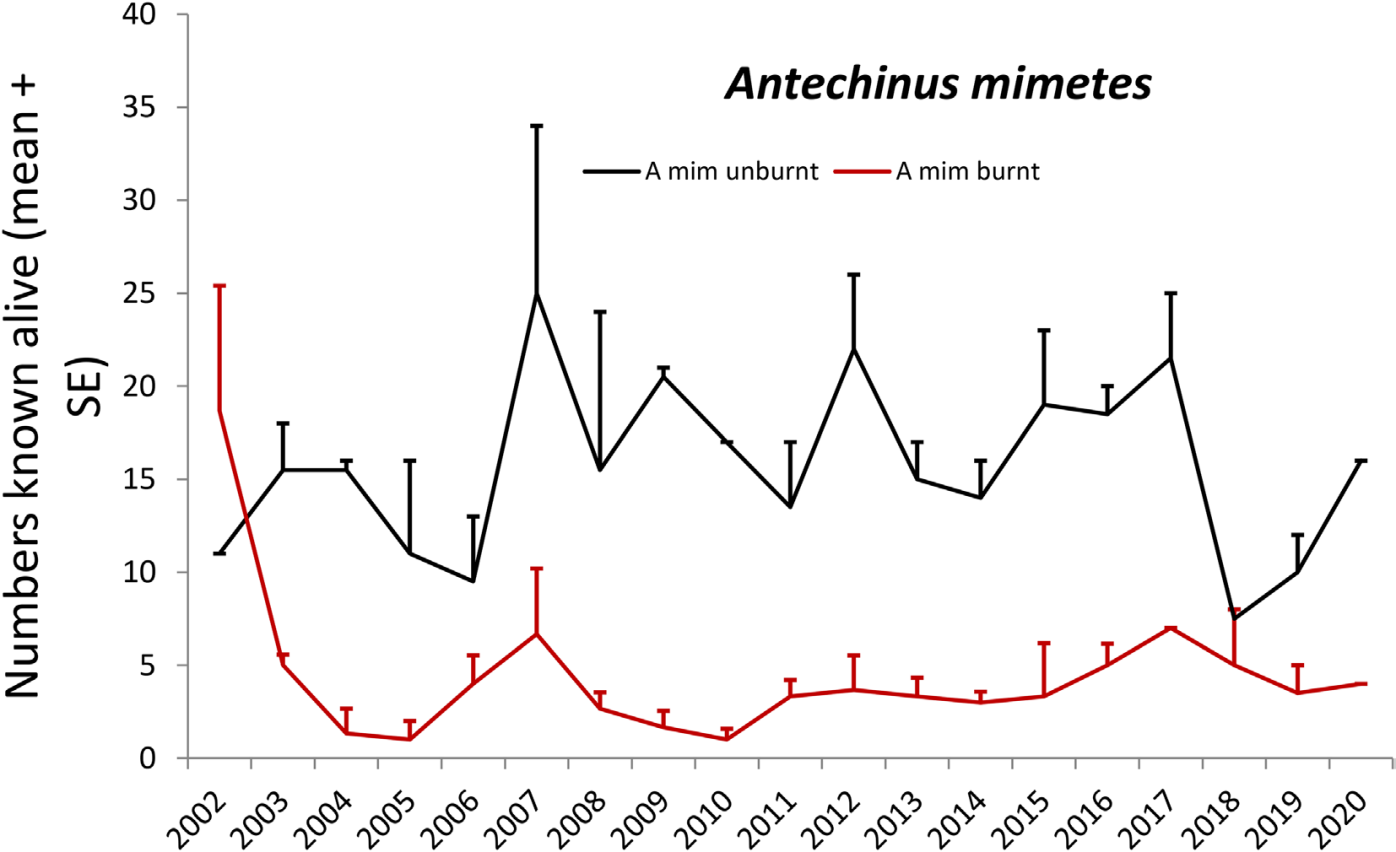
Numbers of mainland dusky antechinus *Antechinus mimetes* known alive between 2002 and 2020 at secondary study sites in Kosciuszko National Park, expressed as means + SE for two unburnt sites (Horse Camp and Perisher Creek, black line) and three sites (Rainbow Lake, Whites River and Kerries Ridge, red line) that burnt in early 2003.

Populations of the three species fluctuated between 0 and 37–42 on the main site at Smiggin Holes (Table S2, Figures 3, 4, 5). The lowest numbers occurred post‐fire in 2003–2005, and higher numbers occurred over the first 20 years of the study and again towards the conclusion of trapping in 2020. For *M. fuscus* , interannual population variability was limited until February 1999, with a standard deviation of 32% of the mean for MNA. Numbers fell markedly between 1999 and 2000, recovering briefly before the wildfire (Figure 3). Although the population showed some recovery a decade post‐fire, numbers remained consistently lower than during the early years of the study. Except for a 2‐year peak in MNA pre‐fire, broadly similar trends were seen in *R. fuscipes* (Figure 4). Fluctuations in numbers of *A. mimetes* drove shifts in rate of change in the mid‐late 1980s and over the last 8 years of the study that were greater than changes observed in either of the rodent species (Figure 5). Populations in the secondary study sites were generally lower than at the main site (Table S3), with numbers of each species lower in the burnt than in the unburnt sites (Figures 6, 7, 8).

Red foxes were present throughout the study, with averages of 2.6–32.6 fox tracks and 6–49.6 scats recorded per month of sampling between 1996 and 2004–2005 (Table S4). In general, fox activity was greater in the first 3–4 years of sampling, with 4.2‐fold more scats found per year from 1996 to 1998 than annually from 1999 to 2004, and 3.6‐fold more snow‐tracks recorded annually from 1996 to 1999 than each year between 2000 and 2005. The two methods tracked together over several years, but disparities in 1998 when most scats were collected but moderate numbers of tracks on snow were counted, and in 1999 when high numbers of snow‐tracks were associated with relatively few scats, resulted in weak correspondence between the two index methods of fox activity (*r* = 0.609, *p* = 0.082).

**FIGURE 9:**
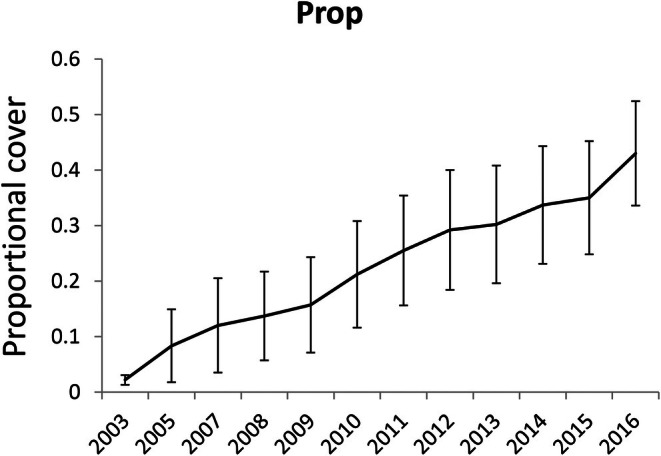
Cover of shrubby vegetation on study sites in Kosciuszko National Park that burnt in a wildfire in early 2003, expressed as the proportion (mean ± SE) of vegetation strikes averaged across height intervals 40 cm to > 120 cm. Grass cover, at 20 cm, averaged 94%–98% in every annual survey and is not included in the figure.

Vegetation, mostly grass, recovered quickly post‐fire, with 98% cover recorded at a height of 20 cm nine months post‐fire. Grass cover remained between 94% and 98% in each annual survey until 2016. Shrubs recovered more slowly (Figure 9), with 50% cover at 40 cm achieved by 2007, 50% cover at 60 cm by 2012 and 49% cover at 80 cm achieved by 2016 (Table S5). Although vegetation appeared to have recovered structurally by 2016, *Eucalyptus niphophila* increased its coverage compared to before the wildfire, as did *Bossiaea foliosa* at higher elevations (unpub. data).

**FIGURE 10:**
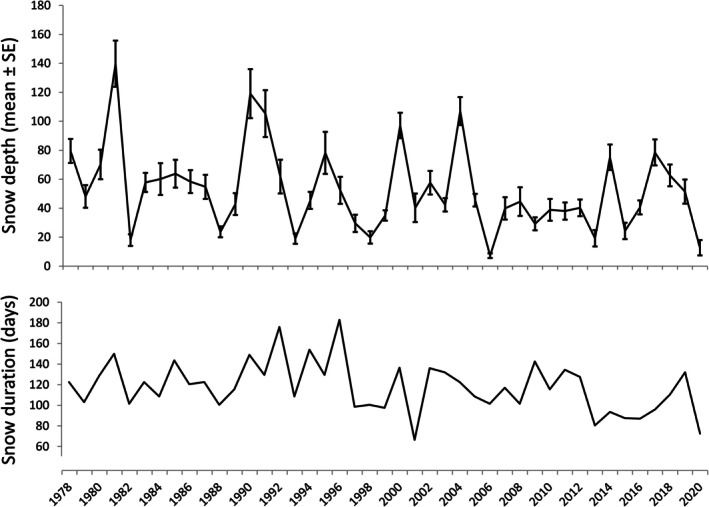
Snow depth (cm, mean per week ± SE) (top panel) and duration of snow cover (days) (bottom panel) from 1978 to 2020 recorded at the Snowy Hydro snow course at Deep Creek, Kosciuszko National Park.

Snow conditions varied dramatically over the study, with mean depth varying from 7.2 cm in 2006 to 139.8 cm in 1981 (grand mean 1978–2020 = 54.5 cm) and snow duration from 66.5 days in 2001 to 183 days in 1996 (grand mean 1978–2020 = 115.1 days). There appeared to be a general downward trend in both variables over the study period (Figure 10).

### 3.1 Hypothesis 1: Populations Will Decline in Years When There Is a Shallow or Transient Snowpack

#### 3.1.1 Main Site

At Smiggin Holes, the timing of the end of the snow season emerged as a significant influence on populations of *M. fuscus* and *A. mimetes* , with late thaws followed by higher numbers of both species in the following summer. However, *R* ^2^ values of 0.14 for *M. fuscus* and 0.24 for *A. mimetes* indicate that additional factors also account for the unexplained variance in MNA (Table S6). The beginning of the snow season and mean snow depth × seasonal duration had no clear effect on MNA for either species, and no variables were significant for *R. fuscipes* (Table S6):

M.fuscus:MNA=−99.01+0.134start snow+0.419*endsnow−6.470cmdays'

R.fuscipes:MNA=11.62+0.020start snow−0.040endsnow+4.691cmdays'

A.mimetes:MNA=−108.14+0.115start snow+0.467*endsnow−5.087cmdays'

Rates of population change (RoC) at Smiggin Holes were not related to snow variables in *M. fuscus* and *A. mimetes* , and showed only a weak association (*R* ^2^ = 0.039) with the product of mean snow depth and duration for *R. fuscipes* . No regression equations for RoC achieved statistical significance (*F*‐values ranged from 0.428–1.831 and associated *p*‐values from 0.168–0.735; Table S6):

M.fuscus:RoC=−52.37+0.011start snow+0.314endsnow−10.139cmdays'

R.fuscipes:RoC=35.11−0.158start snow−0.277endsnow+17.936*cmdays'

A.mimetes:RoC=−47.54+0.049start snow+0.140endsnow+0.631cmdays'

#### 3.1.2 Secondary Sites

At the unburnt secondary sites, no significant relationships were found between the numbers of either species of rodent and snowpack variables (*F*‐values ranged from 0.306–0.773, *p*‐values from 0.527–0.820). The beginning and the end of the snow season were associated negatively with numbers of *A. mimetes* in the following summer (*F*_3,15_ = 8.128, *p* = 0.002). Thus, MNA = 105.18−0.145* (start snow) − 0.26* (end snow) + 1.103 (cm days'). The associated *R* ^2^ values were 0.36 (start snow) and 0.20 (end snow) and 0.23 (cm days'). At the burnt secondary sites a significant regression was obtained for numbers of *M. fuscus* and snowpack variables (*F*_3,14_ = 5.1, *p* = 0.014), but no individual variable was significant (Table S7). No relationships were evident between numbers of *R. fuscipes* and snowpack variables, but the end of the snow season was associated negatively and significantly (*F*_3,14_ = 6.04, *p* = 0.007) with numbers of *A. mimetes* the following year. Thus, MNA = 32.07 + 0.025 (start snow) − 0.10* (end snow) + 1.197 (cm days'); the associated end‐snow *R* ^2^ = 0.51. Rates of population change showed no relationship with snow variables at the secondary sites (*F*‐values ranged from 0.249–1.689, and associated *p*‐values from 0.218–0.861, Table S7).

#### 3.1.3 Combined Main Site and Unburnt Secondary Sites

To further examine declines in small mammal numbers, we used estimates of MNA at the main site up to 2002 (pre‐fire) and averaged estimates of MNA at the unburnt secondary sites from 2003 to identify years over the duration of the study—in the absence of fire—when MNA declined by ≥ 50% from the year prior. For *M. fuscus* , these were 1989, 1991, 2000, 2006, 2010, 2017 and 2019 (Figures 3 and 6). Except for 1991, when mean snow depth in the year prior was 119.1 cm, declines in numbers of *M. fuscus* occurred following years of shallow snow cover (mean depth = 39.44 cm ± 6.63 SE) compared with other years (mean depth = 55.25 cm ± 5.67 SE). Other snowpack variables were similar across decline and non‐decline years for *M. fuscus* . Declines in MNA of ≥ 50% occurred too infrequently in *R. fuscipes* (1980, 2006 and 2017) and *A. mimetes* (2018) to allow meaningful comparisons.

Taken together, these results provide some evidence in support of our first hypothesis: populations of *M. fuscus* declined sharply in some (but not all) years following winters with shallow snow cover. The timing, especially the end of snow cover, was associated with numbers of *M. fuscus* and *A. mimetes* in the following summer or autumn.

### 3.2 Hypothesis 2: Declines Will Be More Severe for *M. fuscus* Than for Other Small Mammals

At Smiggin Holes, an overall difference was found between the regression slope for *R. fuscipes* (*β* = 0.037 ± 0.111 SE) compared with those for *M. fuscus* (*β* = 0.284 ± 0.138) and *A. mimetes* (*β* = 0.383 ± 0.125) for the end of the snow season (*F*_3,27_ = 3.516, *p* = 0.028). No differences were found for the start of the snow season (*F*_3,27_ = 0.659, *p* = 0.585) or the product of mean snow depth and snow duration (*F*_3,27_ = 1.499, *p* = 0.237). ANCOVAs on rate of change were non‐significant for all variables (*F*_3,24_ < 0.85, *p*‐values 0.484–0.779). These results do not support our second hypothesis.

### 3.3 Hypothesis 3: Populations of All Species Will Decline Sharply After Wildfire

Captures of each species declined dramatically at the main site after the wildfire in early 2003, with rates of population change plunging to the lowest levels recorded throughout the study and remaining negative for 1–4 years post‐fire (Figures 3, 4, 5). Population recovery was slowest in *M. fuscus* , with numbers reaching pre‐fire levels only transiently in 2018, 15 years after the wildfire (Figure 3). Recovery began 3 years post‐fire in *R. fuscipes* and *A. mimetes* , with the former species showing sustained recovery almost to pre‐fire levels until the conclusion of sampling in 2020 (Figure 4) and the latter species averaging just 55% of its pre‐fire population levels after initial recovery in 2006 (Figure 5).

At the secondary sites, the numbers of all species declined sharply post‐fire (Figures 6, 7, 8). Populations of *M. fuscus* and *A. mimetes* remained lower in the burnt than in the unburnt sites until sampling concluded in 2020 and showed little sign of recovery ( *M. fuscus* : *F*_1,17_ = 28.84, *p* < 0.001; *A. mimetes* : *F*_1,17_ = 132.3, *p* < 0.001). By contrast, *R. fuscipes* recovered to similar numbers as those in the unburnt sites by 2009, although populations over the entire period 2003–2020 remained ~8% less in the burnt than the unburnt sites (*F*_1,17_ = 1.803, *p* = 0.197). Taken together, these findings support our third hypothesis.

## 4 Discussion

Populations of the three study species showed moderate stability for the first 20 years at our main longitudinal study site, but fluctuated markedly over the next 20 years, providing mixed support for our initial hypotheses. A shallow winter snowpack was followed by sharp declines of *M. fuscus* over the following summer in some years, and early loss of the winter snowpack had a small but negative effect on numbers of *M. fuscus* and *A. mimetes* . These effects were stronger for these two species than for *R. fuscipes* . Our third hypothesis was supported most strongly; populations of all three species declined rapidly to zero soon after wildfire at Smiggin Holes and remained lower in our burnt than unburnt secondary sites for several further years. We discuss these findings below and comment on the implications that may be drawn for management and about the future effects of climate change.

### 4.1 Effects of the Winter Snowpack

Prior to the wildfire at our main study site in 2003, sharp falls to low numbers were observed for *R. fuscipes* between 1979 and 1980 and for both *M. fuscus* and *A. mimetes* between 1998 and 2000–01 (Figures 3, 4, 5). In 1979, mean snow depth (48.1 cm) and duration of cover (103 days) were relatively low (89% of the respective long‐term means), while the years 1997–1999 had mean annual snow depths (19.8–35 cm) that were 36%–64% of the long‐term mean, and annual durations (97.5–100.5 days) that were 85%–87% of the long‐term mean. Although we expected poor snow seasons to be followed by small mammal declines (hypothesis 1), as in species such as Arctic brown lemmings ( *Lemmus trimucronatus* ; Bilodeau et al. 2013), other years with a shallow snowpack (e.g., 1982) or deep snowpack (e.g., 1981) or long or short periods of snow cover (e.g., 149 days in 1990, 66.5 days in 2001), were associated inconsistently with changes in numbers of the study species. Brief or shallow snowpacks occurred in several years after the 2003 wildfire, notably during and after the Millenium Drought (e.g., from 2006–2013 and 2015–2016 mean annual snow depths were 13%–82% of the annual long‐term mean). Low numbers of *M. fuscus* were recorded in many of these years, including in the unburnt secondary sites, providing some evidence that snowpack depth affects this species, but inconsistency in snowpack effects between years suggests that other factors also operate. For example, as judged by scats and tracks in snow, the activity of red foxes was high in 1998 and 1999. Shallow snowpacks in both these years (mean 19.8 cm in 1998; 35 cm in 1999) may have allowed foxes access to the subnivean space, hastening the decline of *M. fuscus* to low numbers in 2000. In the Arctic, deep snow cover reduces predation by Arctic foxes (*Vulpes alopex*) on collared lemmings ( *Dicrostonyx groenlandicus* ; Duchesne et al. 2011). Unfortunately, we have no data on fox activity before 1996 or after 2005 at our sites to assess possible snowpack × fox activity interactions on small mammal declines in these years.

In addition to these results, another aspect of snow cover—the date of the thaw—emerged in our analyses of the main site data as having an unexpected influence on small mammal dynamics. Higher numbers of both *M. fuscus* and *A. mimetes* occurred in summer following years when snow cover had remained late in the preceding spring, notably into the first or second week of October or later. We propose three explanations. First, changes in the social organisation of *M. fuscus* and *A. mimetes* occur in spring that may place animals at greater risk of mortality in the absence of a continuous snowpack. In *M. fuscus* , individuals switch from sharing nests through winter to living solitarily in spring, with both sexes increasing activity and males expanding their home ranges to encompass those of three or more females (Bubela et al. 1991; Bubela and Happold 1993). These changes herald mating in October and the first births of young from late October to early December (Happold 1989, 2011). In *A. mimetes* , increased movements precede mating and male deaths in September; females then maintain exclusive home ranges prior to births in late October (Dickman 1982). These critical life history events may be less disrupted under a snowpack that remains into October than under freezing conditions if the snowpack has gone by early spring. Green (1998), for example, recorded subnivean temperatures as low as −8°C under patchy snow conditions but relatively constant temperatures of −1°C to 0°C when snow cover was intact.

Second, a late ending to the snow season would release small mammals from the thermally protected subnivean space when ambient air temperatures are increasing. Data from Perisher Creek show that mean daily minimum temperatures increase from −5.7°C in July to −2.2°C in September before rising to 0.2°C in October and remain above freezing until April. Mean daily maxima over the same period show similar trends but never fall below 0°C (Bureau of Meteorology 2024). Gradually warming spring temperatures hasten snow melt, but temperature records suggest that warming can occur rapidly at different times in spring, allowing the snowpack to remain intact until at least mid‐October in some years (Bureau of Meteorology 2024). Like many sub‐alpine small mammals (Hoffman 1974; Wunder 1984; Merritt 2010), adult *M. fuscus* and *A. mimetes* have behavioural, physiological and morphological adaptations that permit survival at low temperatures (Happold 1998; Green 2001). However, the smaller size, delayed fat storage and shorter hair in young animals mean that survival prospects of the young should be greater if ambient temperatures are higher when they become independent. Young‐of‐the‐year animals comprise 23%–57% of the summer population in *M. fuscus* and 85% in *A. mimetes* (Happold 2015), emphasising the likely importance of moderate (> 0°C) spring temperatures in promoting population persistence.

**FIGURE 11:**
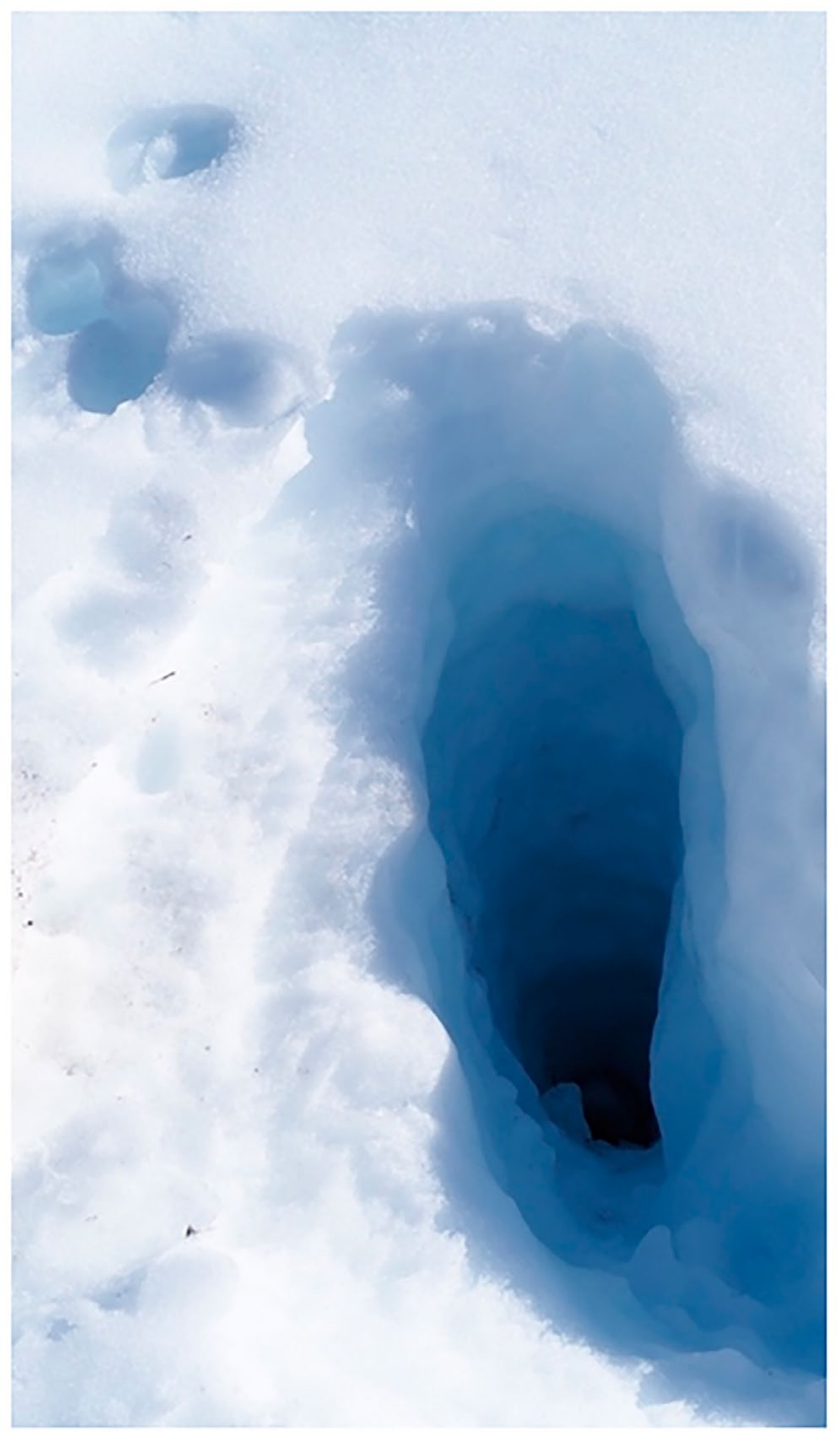
A hole excavated by a European red fox *Vulpes vulpes* in snow to access prey (probably *Mastacomys fuscus* ) in the subnivean space at a depth of > 50 cm in Kosciuszko National Park. Photo: Chris White.

Third, delays in the spring thaw may prolong protection from predation by the red fox. Foxes can dig 55–80 cm into snow to reach small mammals (Figure 11), but this would be energetically expensive and a snowpack of average depth (~55 cm) may provide moderate protection for small mammals in the subnivean space. Nonetheless, when foxes do dig they are most likely to prey upon *M. fuscus* owing to its communal and surface nesting behaviour in winter (Green 2003), and perhaps also *A. mimetes* as its cathemeral activity in the subnivean space would place it at risk of predation at any time (Dickman 1986; Bubela and Happold 1993). Both species are eaten frequently by the red fox in Kosciuszko National Park (Bubela et al. 1998; Green 2003). Between 1996 and 1998, these two species formed 64%–70% by volume of the winter diet of red foxes (Green 2003), with elevated fox activity in 1999 perhaps playing a role in the decline in numbers of *M. fuscus* and *A. mimetes* that we observed the following year (Figures 3 and 5). By October–November alternative prey for the fox, such as insects, become more accessible. In short, extended snow cover in spring may reduce direct predation by the red fox on *M. fuscus* and *A. mimetes* at a key time in the annual life history of these species, and also reduce the time between snow loss and the arrival of abundant alternative prey for the fox that may alleviate predation on the small mammals.

These interpretations of the effect of timing of annual snow loss on small mammal numbers appear plausible, but do not completely explain our results. Thus, *R* ^2^ values (0.14 for *M. fuscus* , 0.24 for *A. mimetes* for the MNA results) indicate that factors in addition to the timing of the thaw influence animal numbers (e.g., disturbance from nearby ski resorts, food, shelter), and analyses based on rate of change failed to support the MNA results. We also found contrary results in the secondary sites, where the beginning and end of the snow season were associated negatively with numbers of *A. mimetes* in the following summer, while *R. fuscipes* showed no clear or consistent responses to snowpack conditions at any sites.

With respect to *A. mimetes* in the unburnt secondary sites, individual site data suggested a contrary result for Horse Camp. Simple correlations between annual MNA for *A. mimetes* at Horse Camp and the beginning and end of snow each year revealed more strongly negative associations (*r* = −0.567 and *r* = −0.492, respectively) than at Perisher Creek (*r* = −0.341 and *r* = −0.177, respectively). A negative relationship between the start of the snow season and summer populations of *A. mimetes* could arise from animals achieving greater body mass and higher survival with the early arrival of snow cover (Green 2001), in turn boosting the reproductive success of overwintering adults and their production of young. An early end to the snow season similarly could lead to greater summer numbers of *A. mimetes* if snow melt increased the access of animals to invertebrate prey. This is plausible, as Horse Camp is at a lower altitude (1540 m) than any of our other study sites so that invertebrate activity and temperatures here should increase earlier in spring than at higher altitudes (Dickman et al. 1983). Our ostensibly contrary results for the unburnt secondary sites may therefore represent differential effects of the timing of the thaw with altitude, at least for *A. mimetes* : early spring loss of snow at lower altitudes (< 1600 m) allows increased invertebrate activity, whereas a later‐lying snowpack at colder, higher altitudes (> 1600 m) maintains the moderate conditions of the subnivean space until temperatures warm sufficiently later in spring for prey activity to increase. The negative association in the burnt secondary sites between the spring thaw and summer numbers of *A. mimetes* is more difficult to interpret, but may reflect reduced foraging opportunities in the narrow subnivean space post‐fire (Green and Sanecki 2006).

The inter‐annual fluctuations in the snowpack and climatic conditions had no clear effects on *R. fuscipes* , a result perhaps explicable by several aspects of the biology of this species. Thus, individuals construct convoluted tunnels descending to 30–45 cm below the soil surface (Warneke 1971), with some alpine nests located at depths of > 78 cm. Nests at this depth, and communal nesting in winter (Woodside 1983), provide thermally buffered retreats that reduce dependence on an intact snowpack. *Rattus fuscipes* also is a dietary generalist, consuming leaves of grasses, dicotyledonous herbs and shrubs, fungi, some invertebrates, fruits and seeds (Stewart 1979; Carron et al. 1990; Vernes et al. 2015). At least some of these foods would be available at all times irrespective of the presence of a snowpack; indeed, Carron et al. (1990) found no specific relationship between dietary changes in this species and the beginning or end of the snow season. Third, key changes in the social organisation of *R. fuscipes* —female spacing, increased movements and mating—occur independently of snow cover in November, with the first births occurring in December (Happold 1989, 2015). Unlike in *M. fuscus* and *A. mimetes* , when between‐year variation in numbers is greatest in spring, interannual variation in numbers of *R. fuscipes* is greatest in autumn due to increased adult mortality post‐breeding (Robinson 1988; Happold 2015). Finally, high altitude populations of *R. fuscipes* have greater glycolytic capacity, fur thickness, capacity for non‐shivering thermogenesis and energy conservation than populations at low altitude (Stewart 1979; Glanville et al. 2012). These traits probably serve in sub‐alpine areas to further reduce any dependency of *R. fuscipes* on the integrity, or even presence, of the snowpack.

### 4.2 Effects of Wildfire

As predicted by our third hypothesis, all the study species declined after wildfire. At the main study site *M. fuscus* became locally extinct; it was not captured for three years (2005–2007), and had failed to make a sustained recovery by 2020. Both *R. fuscipes* and *A. mimetes* also were locally extirpated on the main site for 1–2 years, but began to recover three years later and ~ 6 years before *M. fuscus* . Populations of all three species fell post‐fire in the secondary sites and remained generally lower in the burnt than in the unburnt sites, with depressions in numbers particularly marked in *M. fuscus* and *A. mimetes* . We posit several explanations.

First, some animals were probably killed during the wildfire, either being burnt or dying by heat or smoke suffocation. At the time of the wildfire in late January and early February most individuals in each population would have been newly‐independent young. These individuals would have had less knowledge of safe areas such as moist boggy sites or creek banks, or access to such areas, and thus may have suffered relatively high mortality. In Nebraska, for example, young harvest mice *Reithrodontomys megalotis* are particularly susceptible to controlled burns owing to their inability to escape the flames (Erwin and Stasiak 1979). We have no information on direct fire‐induced mortality in our study, but no individuals that were present on the main trapping site in December 2002 were recaptured post‐fire.

A further possibility is that animals mostly survived the fire and either emigrated or succumbed soon afterwards. Recent reviews suggest that many animal species have effective fire‐detection and fire‐avoidance behaviours, especially in fire‐prone habitats (Nimmo et al. 2019, 2021), and that direct fire‐induced mortality is usually low (2%–7%, Jolly et al. 2022). In our study, we suggest that emigration of fire‐survivors to unburnt habitat was unlikely. This is because the area of the Australian Alps that burnt was very extensive—1.73 million ha overall (Worboys 2003) including ~70% of the land above 1500 m (Green and Sanecki 2006)—and because post‐fire emigration in previous work on the study species has shown this to be limited (Sutherland 1998; Green and Sanecki 2006; Dickman and Happold 2022). We also recorded no great influx of new animals on our two secondary site grids that remained unburnt in February 2003, as might have been expected if animals displaced from burnt areas were moving across the landscape in search of unburnt refuges.

Several other factors may have depressed animal numbers in the burnt sites: drought, reduced shelter, reduced food and increased predation pressure (Sutherland and Dickman 1999; Whelan et al. 2002). Dense and structually complex heath dominated each of the study grids that burnt in 2003, and this was largely incinerated by the fire's intensity. Although patches of a few square metres of unburnt habitat remained on each site, these would have provided limited shelter and left surviving animals exposed to cold conditions during autumn and with a limited subnivean space in winter. Green and Sanecki (2006) showed that the depth of the subnivean space in wet heath—the habitat preferred by the three study species—reduced from ~20 cm in unburnt habitat to ~4 cm post‐fire, while in other burnt habitats the subnivean space was ~2 cm. Although grass (*Poa* spp.) grew rapidly after the wildfire, it would have provided little shelter for small mammals. The delayed recovery of shrub cover from 2003 (Figure 9) would have slowed recovery of the subnivean space and probably limited population recovery, especially of *M. fuscus* and *A. mimetes* , for several years.

Shrub loss would have also reduced food resources. Although some grasses and insects were present post‐fire (K. Green, G. Sanecki, unpub.), dicotyledonous leaves and stems, seeds, fruits and invertebrates likely became scarce. One shrub, *Bossiaea foliosa* , recovered most quickly post‐fire (M. Shroder, pers. comm.); as this forms a dense canopy it suppresses the growth of grasses and herbs that are consumed by *M. fuscus* , in particular. Pyrophilous fungi grew and fruited after the fire (McMullan‐Fisher et al. 2011) and may have been exploited by *R. fuscipes* , but fungal growth—like vegetative growth—was likely limited by colder temperatures in autumn. The depletion of green plant material would have led also to reduced invertebrate activity. As this activity declines in many high altitude environments over winter (Sømme 1997; Danks 2004; Buckley et al. 2024), invertebrates may have been scarce over the first winter post‐fire, depriving *A. mimetes* of its principal prey and *R. fuscipes* of a secondary food resource. Winter is a time of food scarcity for small mammals in montane habitats (Schweiger and Boutin 1995; Banks and Dickman 2000; Prevedello et al. 2013); with wildfire depleting food resources further, food shortage probably contributed to local extinctions and then low numbers post‐fire of all the study species.

Dry conditions during the Millenium Drought, which began 2 years before the wildfires, likely exacerbated the extent and severity of the fires. Reduced rainfall during the drought would have slowed the recovery of both shrub cover and food resources, compounding the effects of the wildfire on the study species. As noted by Crowther et al. (2018), linkages between climatic conditions, fire and mammal assemblages can be strong and emphasise a need for long‐term research to disentangle the effects of these factors on wildlife.

### 4.3 Effects of Predation

Predation likely also depleted the numbers of post‐fire survivors. Loss of shrub cover would have increased the exposure of surface‐active animals to avian and mammalian predators, notably the red fox and perhaps feral cats ( *Felis catus* ; Watson et al. 2025). Our indices of fox activity fluctuated over the 3 years before and 3 years after the wildfire. However, the reduction in small mammal numbers post‐fire means that *per capita* predation would have been intense; indeed, Green and Sanecki (2006) noted an immediate and marked increase in the proportion of fox scats collected in burnt areas that contained mammalian hair. The nexus between fire, reduced food and cover resources, and predation has been explored recently (Hradsky et al. 2017; Geary et al. 2020; Doherty et al. 2022), and has led to recognition that these factors in combination can cause local exinction of prey populations. Attempts to disentangle these factors by manipulating fire, predator density, prey shelter and food indicate that predation often has negative effects on prey populations post‐fire (Conner et al. 2011; Morris, Hostetler, Conner, and Oli 2011; Morris, Hostetler, Oli, and Conner 2011), but the relative importance of these factors varies between species, times and places (Sutherland 1998; Karmacharya et al. 2013; Watchorn et al. 2024).

In our study, *M. fuscus* showed the slowest post‐fire recovery of the three study species. This may reflect the slow recovery of diverse shrubby vegetation and the cover that this provides from foxes—for which *M. fuscus* is preferred prey (Green 2002, 2003)—as well as support of the snowpack to create an effective subnivean space. At altitudes of 1030–1555 m in northern Kosciuszko National Park, Schulz et al. (2024) reported a rapid recovery of *M. fuscus* with regrowth of vegetation after wildfires in 2019–2020; one year post‐fire the species occurred in just 8% of formerly occupied sites, and 3 years post‐fire there was 66% occupancy. In contrast to *M. fuscus* , *A. mimetes* and especially *R. fuscipes* showed faster recoveries post‐fire. *Antechinus mimetes* features prominently in the diet of the red fox in sub‐alpine areas (Green and Osborne 1981), but its deeper burrows may facilitate increased survival and its greater reproductive potential should help numbers to rebuild quickly (Happold 2011, 2015). At lower altitudes (800–850 m), Dickman and Happold (2022) attributed post‐fire depression in numbers of *A. mimetes* to depleted populations of invertebrate prey, and recovery a year later to the regeneration of those prey. We did not sample invertebrates here, but suggest the slower (3‐year) recovery of *A. mimetes* compared with that documented by Dickman and Happold (2022) reflects slower growth of invertebrate populations at higher altitudes.

The post‐fire recovery of *R. fuscipes* was the fastest of the study species. Like *A. mimetes* , this species uses burrows for shelter and has high reproductive potential (Happold 2011, 2015), but unlike either the marsupial or *M. fuscus* it is preyed upon less frequently by red foxes (Green 2003) and is able to switch its diet to include whatever green plant, fungal or invertebrate resources are available (Dickman and Happold 2022; Kanishka et al. 2025). Populations of *R. fuscipes* often fall to low numbers post‐fire (e.g., Fox 1982; Lindenmayer et al. 2008; Recher et al. 2009), but the ‘fast’ life history and flexible resource use of this species may allow relatively fast recovery, especially if other factors such as predation or drought do not prolong population troughs (Crowther et al. 2018; Dickman and Happold 2022).

For recovery to happen at all post‐fire, colonising animals need to come from somewhere. Recovery of small mammals often begins with individuals that have survived a fire in situ, perhaps in burrows or unburnt vegetation (Banks et al. 2011, 2017; Hale et al. 2022). This plausibly occurred in our secondary study sites where, except for one year when no *M. fuscus* was captured (2004), small numbers of animals were always present and could have served as foci for later population expansion. However, this explanation is less likely on the main study site. Here, *R. fuscipes* was not recorded in one annual trapping session, *A. mimetes* over two consecutive sessions and *M. fuscus* between 2005 and 2007. The main study grid was burnt severely and, except for some unburnt vegetation near the access road, probably provided immigrants with few resources to facilitate settlement until vegetation began to re‐establish. The slower recovery of *M. fuscus* and *A. mimetes* than of *R. fuscipes* may reflect interspecific differences in dispersal distance, and also that dispersal in *M. fuscus* and *A. mimetes* was retarded by fox or cat predation, or disturbance from the ski resort. These two species are the most frequently preyed upon by red foxes in Kosciuszko, with fox predation also disrupting dispersal of *M. fuscus* between habitat patches (O'Brien et al. 2008). By contrast, *R. fuscipes* is preyed upon less frequently; individuals can recognise and avoid odour‐based cues to fox presence and, while foxes can depress the survival of *R. fuscipes* , the impacts of foxes on population size of this prey species appear negligible (Kovacs et al. 2012).

### 4.4 Limitations of the Study

Our primary longitudinal results derive from a relatively small study site. Although shorter time series from our secondary study sites suggest that our main site results broadly reflect the dynamics of small mammals in sub‐alpine wet heath communities, additional sites would have helped to confirm this and also facilitated manipulative approaches to hypothesis‐testing. In the Canadian Arctic, for example, Bilodeau et al. (2012) used snow fences to enhance snow cover at experimental sites and found that a deeper snowpack increased the densities of winter nests of lemmings, but did not affect demographic responses, compared with control sites. Despite our use of a single main site, the 43‐year time series this has yielded on small mammals is nonetheless the longest dataset for any sub‐alpine environment in the southern hemisphere, and it will serve as an increasingly valuable baseline in future.

## 5 Conclusions and Implications for Management

Warming trends in many alpine and sub‐alpine regions are affecting snowpacks and the subnivean spaces that ameliorate overwinter conditions for small mammals, and are increasing the frequency and severity of wildfires. Our results suggest that early loss of the snowpack in spring results in increased exposure to cold temperatures at a critical time in the life history of two of our study species ( *M. fuscus* and *A. mimetes* ), and may also elevate the impacts of invasive predators before alternative prey become available. A shallow snowpack can also lead to declines in numbers of *M. fuscus* . Wildfire may kill animals quickly or lead, with drought, to increased mortality in the post‐fire environment due to the removal of food sources and vegetative cover, as well as to increased predation. Animals that persist post‐fire may assist population recovery as vegetation regrows, but if fires are extensive and leave few survivors over large areas, population recovery will be retarded and occur only by immigration. For threatened species such as *M. fuscus* and common but localised species such as *A. mimetes* that face selective predation by the red fox, populations will become increasingly depleted. The effects of reduced snowpack, wildfires and droughts are likely to be synergistic and could result in sudden declines of small mammals that result from the crossing of survival thresholds rather than from trends of decreasing numbers.

Options for management of biota in high altitude environments have been much discussed globally (e.g., IPCC 2023), but in the Australian Alps we argue that research and monitoring must be expanded to track and better understand biotic—environmental changes. Populations and communities of at‐risk species should be identified and strategies developed to mitigate the impacts of threats such as wildfires and incursions by invasive species. A Biodiversity Bureau has been proposed recently to integrate such environmental monitoring data and tools and support decision‐makers in conserving natural systems (Dickman 2021). We suggest that a similar but local body for the Australian Alps be established to coordinate and develop local conservation and management plans for taxa and ecosystems that are at most risk of collapse.

## Author Contributions

**Ken P. Green:** conceptualization (equal), data curation (lead), formal analysis (equal), funding acquisition (supporting), investigation (equal), methodology (equal), project administration (equal), resources (equal), supervision (equal), validation (equal), visualization (supporting), writing – original draft (supporting), writing – review and editing (equal). **David C. D. Happold:** conceptualization (equal), data curation (supporting), formal analysis (supporting), funding acquisition (lead), investigation (equal), methodology (equal), project administration (equal), resources (equal), supervision (equal), validation (supporting), visualization (supporting), writing – original draft (supporting), writing – review and editing (equal). **Glenn M. Sanecki:** conceptualization (equal), data curation (supporting), formal analysis (supporting), funding acquisition (supporting), investigation (equal), methodology (supporting), resources (equal), supervision (supporting), validation (supporting), visualization (supporting), writing – original draft (supporting). **Christopher R. Dickman:** conceptualization (equal), data curation (supporting), formal analysis (equal), investigation (supporting), methodology (supporting), resources (equal), validation (equal), visualization (lead), writing – original draft (lead), writing – review and editing (equal).

## Funding

This work was supported by M.A. Ingram Trust, Faculty Research Trust of the Australian National University and Ethel Mary Read Research Grant Fund of the Royal Zoological Society of New South Wales.

## Ethics Statement

All research was carried out with approval by the Animal Ethics Committee of the Australian National University (approval nos F.BTZ.56.95 and F.BTZ65.97) and under scientific licences granted by the New South Wales National Parks and Wildlife Service (Licence nos A125 and C275).

## Conflicts of Interest

The authors declare no conflicts of interest.

## Supporting information


**Table S1:** Snow variables.

**Table S2:** MNA main site.

**Table S3:** MNA secondary sites.

**Table S4:** Fox activity.

**Table S5:** Vegetation cover.

**Table S6:** Main site regressions.

**Table S7:** Secondary site regressions.

**Data S1:** ece373525‐sup‐0008‐DataS1.docx.

## Data Availability

All required data are uploaded as Supporting Information.
