# Supplementary material for: Fire and Snow: Effects of Snowpack Variation and Wildfire on Small Mammal Dynamics in Sub‐Alpine Habitats
Source: Ecol Evol. 2026 Apr 20;16(4):e73525. doi: 10.1002/ece3.73525 (PMC13095868; doi:10.1002/ece3.73525)
Supplement: Supplementary file 6 — Table S6: Main site regressions. [file ECE3-16-e73525-s008.docx]

**Table S6 Main site (Smiggin Holes) regression analyses on numbers (MNA) and rate of change**

**Response variable: *Mastacomys fuscus* numbers 1978-2020**

**ANOVA *F***_3,29_ = 2.390 *P* = 0.089

N = 33 Multiple *R* = 0.445 Multiple *R*^2^ (adj) = 0.115

**Parameter Estimate s.e. *t* *P* *R*^2^**

Constant -99.005 42.889 -2.308 0.028

Start 0.134 0.0948 1.409 0.170 0.040

End 0.419 0.196 2.138 0.041 0.139

cm.days' -6.470 8.188 -0.790 0.436 0.045

**Response variable: *Rattus fuscipes* numbers 1979-2020**

**ANOVA *F***_3,27_ **=** 0.251 *P* = 0.860

N = 31 Multiple *R* = 0.165 Multiple *R*^2^ (adj) = -0.081

**Parameter Estimate s.e. *t* *P* *R*^2^**

Constant 11.623 35.747 0.325 0.748

Start 0.020 0.0811 0.249 0.805 0.009

End -0.040 0.167 -0.239 0.813 0.004

cm.days' 4.691 7.397 0.634 0.531 0.021

**Response variable: *Antechinus mimetes* numbers 1979-2020**

**ANOVA *F***_3,27_ **=** 3.633 *P* = 0.025

N = 31 Multiple *R* = 0.536 Multiple *R*^2^ (adj) = 0.208

**Parameter Estimate s.e. *t* *P* *R*^2^**

Constant -108.14 39.489 -2.739 0.0108

Start 0.115 0.090 1.280 0.211 0.034

End 0.467 0.184 2.533 0.017 0.243

cm.days' -5.087 8.171 -0.623 0.539 0.106

**Response variable: *Mastacomys fuscus* rate of change 1978-2020**

N = 29 Multiple *R* = 0.311 Multiple *R*^2^ (adj) = -0.012

**ANOVA *F***_3,25_ = 0.893 *P* = 0.458

**Parameter Estimate s.e. *t* *P* *R*^2^**

Constant -52.367 42.608 -1.229 0.231

Start 0.011 0.097 0.101 0.914 0.003

End 0.314 0.195 1.607 0.121 0.048

cm.days' -10.139 9.099 -1.114 0.276 <0.001

**Response variable: *Rattus fuscipes* rate of change 1979-2020**

N = 28 Multiple *R* = 0.432 Multiple *R*^2^ (adj) = 0.085

**ANOVA *F***_3,24_ = 1.831 *P* = 0.168

**Parameter Estimate s.e. *t* *P* *R*^2^**

Constant 35.107 39.644 0.886 0.385

Start -0.158 0.090 -1.753 0.092 0.038

End -0.277 0.182 -1.526 0.140 0.001

cm.days' 17.936 8.598 2.086 0.048 0.039

**Response variable: *Antechinus mimetes* rate of change 1979-2020**

N = 28 Multiple *R* = 0.225 Multiple *R*^2^ (adj) = -0.068

**ANOVA *F***_3,24_ =0.428 *P* = 0.735

**Parameter Estimate s.e. *t* *P* *R*^2^**

Constant -47.542 45.484 -1.045 0.306

Start 0.049 0.103 0.475 0.639 0.012

End 0.140 0.209 0.672 0.508 0.039
