# Supplementary material for: Fire and Snow: Effects of Snowpack Variation and Wildfire on Small Mammal Dynamics in Sub‐Alpine Habitats
Source: Ecol Evol. 2026 Apr 20;16(4):e73525. doi: 10.1002/ece3.73525 (PMC13095868; doi:10.1002/ece3.73525)
Supplement: Supplementary file 7 — Table S7: Secondary site regressions. [file ECE3-16-e73525-s001.docx]

**Table S7 Secondary site regression analyses on numbers (MNA) and rate of change**

**Response variable: *Mastacomys fuscus* numbers 2002-2020 UNBURNT SITES**

N = 19 Multiple *R* = 0.240 Multiple *R*^2^ (adj) = -0.131

**ANOVA *F***_3,15_ = 0.306 *P* = 0.820

**Parameter Estimate s.e. *t* *P* *R*^2^**

Constant -1.072 19.189 -0.056 0.956

Start 0.030 0.0322 0.940 0.362 0.041

End 0.024 0.0839 0.281 0.783 0.002

cm.days' -1.596 3.099 -0.515 0.614 0.002

**Response variable: *Rattus fuscipes* numbers 2002-2020 UNBURNT SITES**

N = 19 Multiple *R* = 0.366 Multiple *R*^2^ (adj) = -0.039

**ANOVA *F***_3,15_ = 0.773 *P* = 0.527

**Parameter Estimate s.e. *t* *P* *R*^2^**

Constant 18.186 37.351 0.487 0.633

Start -0.054 0.063 -0.859 0.404 0.004

End -0.101 0.163 -0.620 0.545 0.014

cm.days' 8.611 6.033 1.427 0.174 0.086

**Response variable: *Antechinus mimetes* numbers 2002-2020 UNBURNT SITES**

N = 19 Multiple *R* = 0.787 Multiple *R*^2^ (adj) = 0.543

**ANOVA *F***_3,15_ = 8.128 *P* < 0.01

**Parameter Estimate s.e. *t* *P* *R*^2^**

Constant 105.180 23.178 4.538 <0.001

Start -0.145 0.039 -3.717 <0.02 0.357

End -0.260 0.101 -2.563 0.022 0.200

cm.days' 1.103 3.744 0.295 0.772 0.230

**Response variable: *Mastacomys fuscus* numbers 2003-2020 BURNT SITES**

N = 18 Multiple *R* = 0.723 Multiple *R*^2^ (adj) = 0.420

**ANOVA *F***_3,14_ = 5.101 *P* = 0.014

**Parameter Estimate s.e. *t* *P* *R*^2^**

Constant 15.327 4.858 3.155 0.007

Start 0.002 0.0102 0.242 0.812 0.008

End -0.037 0.0216 -1.733 0.105 0.453

cm.days' -1.087 0.867 -1.254 0.231 0.373

**Response variable: *Rattus fuscipes* numbers 2003-2020 BURNT SITES**

N = 18 Multiple *R* = 0.279 Multiple *R*^2^ (adj) = -0.120

**ANOVA *F***_3,14_ = 0.393 *P* = 0.760

**Parameter Estimate s.e. *t* *P* *R*^2^**

Constant -25.673 39.050 -0.657 0.522

Start 0.056 0.082 0.689 0.502 0.032

End 0.122 0.173 0.706 0.491 0.040

cm.days' -1.263 6.970 -0.181 0.859 0.034

**Response variable: *Antechinus mimetes* numbers 2003-2020 BURNT SITES**

N = 18 Multiple *R* = 0.751 Multiple *R*^2^ (adj) = 0.471

**ANOVA *F***_3,14_ = 6.040 *P* = 0.008

**Parameter Estimate s.e. *t* *P* *R*^2^**

Constant 32.066 9.6414 3.326 0.005

Start 0.025 0.020 1.263 0.227 0.049

End -0.101 0.043 -2.363 0.033 0.514

cm.days' -1.197 1.721 -0.695 0.498 0.171

**Response variable: *Mastacomys fuscus* rate of change 2002-2020 UNBURNT SITES**

N = 18 Multiple *R* = 0.293 Multiple *R*^2^ (adj) = -0.110

**ANOVA *F***_3,14_ = 0.440 *P* = 0.728

**Parameter Estimate s.e. *t* *P* *R*^2^**

Constant 13.033 29.920 0.436 0.670

Start -0.060 0.052 -1.144 0.272 0.074

End -0.040 0.120 -0.333 0.744 <0.001

cm.days' 1.870 4.504 0.415 0.684 <0.001

**Response variable: *Rattus fuscipes* rate of change 2002-2020 UNBURNT SITES**

N = 18 Multiple *R* = 0.457 Multiple *R*^2^ (adj) = 0.039

**ANOVA *F***_3,14_ = 1.232 *P* = 0.335

**Parameter Estimate s.e. *t* *P* *R*^2^**

Constant 59.03 42.529 1.388 0.187

Start -0.001 0.074 -0.015 0.988 0.001

End -0.122 0.171 -0.714 0.487 0.125

cm.days' -6.973 6.402 -1.089 0.294 0.177

**Response variable: *Antechinus mimetes* rate of change 2002-2020 UNBURNT SITES**

N = 18 Multiple *R* = 0.452 Multiple *R*^2^ (adj) = 0.034

**ANOVA *F***_3,14_ = 1.201 *P* = 0.345

**Parameter Estimate s.e. *t* *P* *R*^2^**

Constant -33.032 47.898 -0.690 0.502

Start 0.145 0.084 1.739 0.104 0.201

End 0.049 0.193 0.254 0.803 <0.001

cm.days' -0.593 7.2103 -0.082 0.936 0.027

**Response variable: *Mastacomys fuscus* rate of change 2003-2020 BURNT SITES**

N = 17 Multiple *R* = 0.458 Multiple *R*^2^ (adj) = 0.029

**ANOVA *F***_3,13_ = 1.152 *P* = 0.365

**Parameter Estimate s.e. *t* *P* *R*^2^**

Constant -8.776 7.381 -1.189 0.256

Start 0.002 0.016 0.113 0.912 0.043

End 0.014 0.030 0.451 0.659 0.090

cm.days' 1.308 1.242 1.053 0.311 0.197

**Response variable: *Rattus fuscipes* rate of change 2003-2020 BURNT SITES**

N = 17 Multiple *R* = 0.233 Multiple *R*^2^ (adj) = -0.164

**ANOVA *F***_3,13_ = 0.249 *P* = 0.861

**Parameter Estimate s.e. *t* *P* *R*^2^**

Constant 25.011 48.333 0.517 0.614

Start -0.082 0.107 -0.763 0.459 0.047

End -0.064 0.197 -0.326 0.750 0.003

cm.days' 1.474 8.131 0.181 0.859 0.012

**Response variable: *Antechinus mimetes* rate of change 2003-2020 BURNT SITES**

N = 17 Multiple *R* = 0.523 Multiple *R*^2^ (adj) = 0.114

**ANOVA *F***_3,13_ = 1.689 P = 0.218

**Parameter Estimate s.e. *t* *P* *R*^2^**

Constant -29.035 14.323 -2.027 0.064

Start 0.025 0.032 0.782 0.448 0.067

End 0.081 0.059 1.385 0.189 0.188

cm.days' 0.849 2.410 0.352 0.730 0.171
