# Supplementary material for: Fire and Snow: Effects of Snowpack Variation and Wildfire on Small Mammal Dynamics in Sub‐Alpine Habitats
Source: Ecol Evol. 2026 Apr 20;16(4):e73525. doi: 10.1002/ece3.73525 (PMC13095868; doi:10.1002/ece3.73525)
Supplement: Supplementary file 8 — Data S1: ece373525‐sup‐0008‐DataS1.docx. [file ECE3-16-e73525-s007.docx]

**Supporting information: Metadata for the supplementary materials for:**

'Fire and snow: effects of snowpack variation and wildfire on small mammal dynamics in sub-alpine habitats' by K. Green, D.C.D. Happold, G.M. Sanecki, C.R. Dickman.

**Supplementary materials include:**

**1)** Supporting information metadata (this file) and Tables S1 - S7.

**2)** **Table S1: Snow variables.** Column headings describe snowpack variables each year from 1977 to 2020 that were used in analyses. All data were recorded by, or derived from, the snow depth records obtained every week throughout the winter at the Snowy Hydro snow course at Deep Creek, Kosciuszko National Park, as on accessed 3 February 2024 from: https://www.snowyhydro.com.au/generation/live-data/snow-depths/

- **Year:** 1977 - 2020.
- **Snow depth max:** The maximum snow depth (cm) recorded in any week throughout each annual snow season.
- **Snow depth mean:** The mean snow depth (cm) calculated for each week of the annual snow season from when snow first fell to when all snow had gone.
- **Depth SD:** The standard deviation of the weekly snow depth mean data.
- **Depth CV:** The coefficient of variation of the weekly snow depth mean data.
- **Snow start:** The beginning of the snow season each year, taken as the date midway between the last zero reading at Deep Creek and the first reading of snow depth leading to the establishment of a continuous winter snow pack, recorded as the Julian day (number of days from 1 January).
- **Snow end:** The end of the snow season each year, taken as the date when the snow course at Deep Creek was recorded as clear of snow, recorded as the Julian day (number of days from 1 January).
- **Duration:** The number of days between the snow start and snow end each year.
- **Duration - weeks with no snow:** The number of days between the snow start and snow end each year, excluding any periods when snow cover was recorded as zero.
- **Mean depth x Duration - weeks with no snow:** The product of Snow depth mean and Duration - weeks with no snow.
- **Mean depth x Duration - weeks with no snow: log_10_:** The product of Snow depth mean and Duration - weeks with no snow transformed to log_10_.

**3) Table S2. MNA Main Site:** Estimates of the minimum numbers of animals (*Mastacomys fuscus*, *Rattus fuscipes* and *Antechinus mimetes*) known to be alive on the trapping grid at the main study site at Smiggin Holes based on estimates made from sampling in February or April each year. No trapping was undertaken in the year 1981, 1992–1995 and 1997; blank cells represent data absence.

**4) Table S3. MNA Secondary Sites:** Estimates of the minimum numbers of animals (*Mastacomys fuscus*, *Rattus fuscipes* and *Antechinus mimetes*) known to be alive on five secondary trapping sites based on estimates made from sampling in February or April each year. Three sites (Rainbow Lake, Whites River and Kerries Ridge) were burnt by wildfire in early 2003, and two sites (Horse Camp and Perisher Creek) remained unburnt. Means for MNA for the burnt and unburnt sites were used in analyses; blank cells represent data absence.

**5) Table S4. Fox activity:** 'Fox scats' represent average numbers of fox scats collected per month between 1996 and 2004, and 'Fox tracks' represent average numbers of fox tracks counted during months with snow cover between 1996 and 2005.

**6) Table S5. Vegetation cover:** Cells show proportional cover of vegetation at different heights (20 cm to >120 cm) averaged across study grids that were burnt by wildfire in early 2003. Proportions were derived from strikes of vegetation on a measuring pole (presence = 1, absence = 0). Annual mean and standard errors are shown for proportional cover estimates from 2003 to 2016 and represent estimates of shrubby cover. Records for vegetation cover at 20 cm (grey shaded bar) represented grass and are excluded from mean ± SE cover estimates.

**7) Table S6. Main site regressions:** Model results and summary statistics for regression analyses of MNA and rate of change data for *Mastacomys fuscus*, *Rattus fuscipes* and *Antechinus mimetes* against explanatory variables Snow start, Snow end and Mean depth x Duration - weeks with no snow: log_10_ based on data from the main study site at Smiggin Holes, 1978–2020. Statistically significant (α ≤ 0.05) model and parameter values are shown in red.

**8) Table S7. Secondary site regressions:** Model results and summary statistics for regression analyses of MNA and rate of change data for *Mastacomys fuscus*, *Rattus fuscipes* and *Antechinus mimetes* against explanatory variables Snow start, Snow end and Mean depth x Duration - weeks with no snow: log_10_ based on data from secondary study sites, 2002–2020. Regressions were computed separately for burnt (Rainbow Lake, Whites River and Kerries Ridge) and unburnt (Horse Camp and Perisher Creek) secondary sites using mean MNA and rate of change data from these sites. Regressions for the burnt sites used data from 2003–2020, omitting data from 2002 because these sites had not then burnt. Statistically significant (α ≤ 0.05) model and parameter values are shown in red.
